# Decoding material-specific memory reprocessing during sleep in humans

**DOI:** 10.1038/ncomms15404

**Published:** 2017-05-17

**Authors:** M. Schönauer, S. Alizadeh, H. Jamalabadi, A. Abraham, A. Pawlizki, S. Gais

**Affiliations:** 1 Medical Psychology and Behavioral Neurobiology, Eberhard Karls Universität Tübingen, Silcherstr. 5, Tübingen 72076, Germany; 2 Bernstein Center for Computational Neuroscience, LMU München, Großhadernerstr. 2, Planegg-Martinsried 82152, Germany; 3 Department of Psychology, LMU München, Leopoldstr. 13, München 80802, Germany

## Abstract

Neuronal learning activity is reactivated during sleep but the dynamics of this reactivation in humans are still poorly understood. Here we use multivariate pattern classification to decode electrical brain activity during sleep and determine what type of images participants had viewed in a preceding learning session. We find significant patterns of learning-related processing during rapid eye movement (REM) and non-REM (NREM) sleep, which are generalizable across subjects. This processing occurs in a cyclic fashion during time windows congruous to critical periods of synaptic plasticity. Its spatial distribution over the scalp and relevant frequencies differ between NREM and REM sleep. Moreover, only the strength of reprocessing in slow-wave sleep influenced later memory performance, speaking for at least two distinct underlying mechanisms between these states. We thus show that memory reprocessing occurs in both NREM and REM sleep in humans and that it pertains to different aspects of the consolidation process.

## Abstract (web summary)

Neuronal learning activity is reactivated during sleep but the dynamics of this reactivation in humans are still poorly understood. Here the authors show that memory processing occurs during all stages of sleep in humans but that reprocessing of memory content in REM and non-REM sleep has different effects on later memory performance.

Sleep helps us retain new memories^1^,^2^. A reactivation of newly encoded memory traces in the sleeping brain is thought to underlie this effect. Replay of learning-related neuronal firing patterns has been observed in single-cell recordings of the hippocampus and neocortex in animals^3^,^4^,^5^,^6^. Importantly, this sleep-dependent activation of neurons has recently been shown to promote synaptic plasticity^7^. Reactivation of neuronal ensembles involved in motor learning is associated with changes in the task-related spiking behaviour of these neurons in the rodent brain^8^. Furthermore, oscillation related to memory replay during sleep have been linked to greater memory strength and precision in rats^9^. The dynamics of this memory trace reactivation in humans, however, are still poorly understood. When memory content was associated with auditory or olfactory cues during learning, a re-exposure to these cues during sleep can improve later recall performance^10^,^11^. Moreover, activity on the level of brain areas suggests reactivation during sleep^12^,^13^. It is unclear whether this re-expression of learning-related activity reflects the specific content of a previous learning task. Recent advances in multivariate pattern classification (MVPC) methods have made it possible to investigate covert cognitive processes in continuous brain activity. Using such methods on brain activity measured with functional magnetic resonance imaging (fMRI), Horikawa *et al*.^14^ have recently shown that it is possible to decode the content of visual imagery occurring at sleep onset. In the present study, we used MVPC to test whether the human sleep electroencephalogram (EEG) contains information about what has previously been learned and thus indicates reprocessing of memory content.

In our experiment, participants learned pictures of either faces or houses before sleeping in the laboratory for a whole night. During this time, brain activity was recorded using high-density EEG. We then employed MVPC methods to detect information about the previously learned material in electrical brain activity during sleep (Fig. 1, also see Methods section). We investigated continuous sleep EEG instead of evoked activity, because we were specifically interested in spontaneous information processing in sleep. Cued reactivation, which has already been demonstrated in humans with fMRI, shows that stimulus processing in sleep can lead to memory improvement. Previous studies, however, have not shown that such activity actually occurs spontaneously in humans. After demonstrating the existence of such an activity, we were also interested in the time course of memory reprocessing across the night and in sleep-stage-specific activity. It has been discussed previously whether such reactivation occurs during NREM or REM sleep, and both have been implicated in memory reactivation and consolidation^12^,^13^,^15^,^16^. Furthermore, activity that is present only at specific times during the night indicates that the underlying process is related to discrete periods of reprocessing rather than prolonged ongoing activity.

## Results

### Detecting memory reprocessing using MVPC

We tested whether MVPC can decode from the sleeping brain's activity what has been learned beforehand. Instead of looking for a single feature that can distinguish between conditions, MVPC methods take into account and compare the whole temporospatial pattern of activity. Given their multivariate nature, they are more suitable to deal with this kind of high-dimensional problem than is classical statistics, which usually relies on multiple univariate testing. Because EEG activity differs greatly between sleep stages and even more so between sleep and wakefulness, activity cannot be compared directly between these states. We therefore used between-subject analyses to compare recordings from the same sleep state, that is, the classifier was trained and tested on sleep data. If MVPC can determine from the sleep recording which type of visual stimulus a subject has learned before sleep, this implies that stimulus-specific reprocessing of the learned material occurs during sleep.

Our results show that human sleep EEG contains information about which kind of visual stimuli was learned before sleep (Fig. 2a). Classification accuracies for this distinction exceed classification rates expected from chance guessing of the classifier, as determined by randomization statistics, during two of the four 90-min segments (Fig. 2b). Thus the sleep EEG reflects previous learning during these intervals. Moreover, both NREM and REM sleep contain relevant information (Fig. 2a–c).

We used two different approaches to ensure that findings are significant and generalizable. First, we generated randomly labelled data, which, per se, cannot contain any information, and compared the performance of the classifier on these random data with its performance on the original observed data (see Supplementary Fig. 1). This test allows to determine the probability of an outcome by chance given that the data contain no actual information and thus provides exact significance values. Because this process, which repeats the whole analysis for each random iteration, is computationally intensive, we could complete only 1,001 repetitions, which allows significance testing with a lower limit of precision of *P*=0.001. In the case of REM sleep of the second 90-min sleep segment, none of these 1,001 random iterations produced higher classification rates than the real data, thus allowing the conclusion of *P*<0.001.

The second approach to ensure generalizability was to compare classification accuracies of training and validation sets. If accuracy is higher during training than during validation testing, the classifier was overfitted to the training data set and uses random feature characteristics that allow separating classes only in the training data, which are not predictive for new data, and thus cannot be generalized. Ideally, classification rates for the validation data should resemble those for the training data. This shows that the classifier can extract meaningful information from the training set and that the learned pattern can be generalized to new data. It can be seen in Fig. 2b that for data from the first (triangles) and third (squares) 90-min sleep segment training accuracy was low (<0.625), but classification accuracy for the validation set was still worse. Thus EEG from these periods does not seem to contain information pertaining to previous learning experience. On the other hand, EEG from the second (circles) and fourth (stars) 90-min sleep segment consistently shows higher training and validation accuracies and, in some cases, shows nearly perfect generalization between training and validation.

### Relating reprocessing to behavioural memory performance

Participants showed good recognition performance in both the face and house learning conditions (see Supplementary Table 1). We did not observe forgetting across the night. This result is in line with other studies on declarative memory consolidation that have shown stable maintenance of memory performance over sleep but significant decline of memory performance after sleep deprivation or daytime wakefulness^17^,^18^. Memory consolidation, that is, the overnight change in performance, was positively correlated with time spent in sleep stage S4 (*r*_64_=0.254, *P*=0.043; Supplementary Table 2), confirming that sleep was related to the consolidation of this task. We also tested the relation of memory consolidation with the strength of memory reprocessing, which was inferred from the classification probability estimates provided by the classifier. We find that memory reprocessing during slow-wave sleep (SWS) shows a positive relation with memory consolidation (*r*_64_=0.329, *P*=0.008; Supplementary Table 3 and Fig. 3). This correlation remained significant after removing the three most influential values determined by leverage statistics (*r*_61_=0.28, *P*=0.030). Memory reprocessing during sleep stage S2 and REM sleep were not related to memory performance (S2: *r*_64_=0.099, *P*=0.436; REM: *r*_56_=−0.199, *P*=0.142). A regression model including strength of reprocessing in S2, SWS and REM sleep as predictors for memory consolidation found that only reprocessing during SWS correlated significantly with memory consolidation (*β*=0.339, *P*=0.020, explaining 9.7% of the variance), reprocessing in S2 and REM sleep was no significant predictor (S2: *β*=−0.064, *P*=0.656, explaining 0.3% of the variance; REM: *β*=−0.112, *P*=0.436, explaining 1% of the variance). Slopes differed significantly between SWS and REM sleep (strength of reprocessing × sleep stage interaction: *P*=0.008), indicating that memory reprocessing in these sleep stages is differentially related to memory consolidation and could thus have different functions.

We then controlled whether general sleep features such as time spent in deep sleep could possibly account for an increase in both behavioural performance as well as classifiability of the data. Entering strength of reprocessing in SWS and time spent in this sleep stage in a regression model, we found that only strength of reprocessing in SWS was a significant predictor of memory consolidation and explained a larger part of the variance (*β*=0.335, *P*=0.006, explaining 11.2% of the variance), whereas duration of SWS was only marginally significant (*β*=0.214, *P*=0.074, explaining 5.2% of the variance). Strength of reprocessing in SWS was independent of time spent in that sleep stage (*r*_64_=−0.025, *P*=0.423) and the partial correlations support the view that strength of reprocessing in SWS and duration of SWS are independent predictors of overnight memory consolidation (partial correlation with strength of reprocessing during SWS controlling for the duration: *r*_64_=0.342, *P*=0.006; partial correlation with duration of SWS controlling for strength of reprocessing: *r*_64_=0.226, *P*=0.074). Analogous regression analyses for strength of reprocessing and time spent in S2 and REM sleep yielded no significant results, as could be expected from the general lack of association with overnight memory consolidation (all *P*>0.143).

While the proportion of variance in overnight memory consolidation that is explained by memory reprocessing during SWS is low in absolute terms, it should be noted that factors such as alertness or individual differences can introduce considerable variance in memory performance. Classifier performance similarly provides a measure of reprocessing strength that is affected by many sources of between-subject variance as it is estimated based on other participants' sleep EEG characteristics. Despite these difficulties, we demonstrate that memory reprocessing during SWS is significantly related to overnight memory retention, suggesting a robust underlying effect.

### Temporal dynamics of reprocessing

We detected processing of learning material during sleep in the second and fourth 90-min segment of the night (Fig. 2). To investigate this pattern on a more fine-grained scale, we split the night into smaller intervals and analysed the time course of classification accuracy across the night with a resolution of 4.5 min, using the same procedure as above. Again, we find two periods of the night during which brain processing seems to be more strongly related to previous learning, congruent with the time windows reported above. During other periods, no learning-related information was detected (Fig. 4).

### Spatial characteristics of reprocessing and frequency contributions

**Figure 1:**
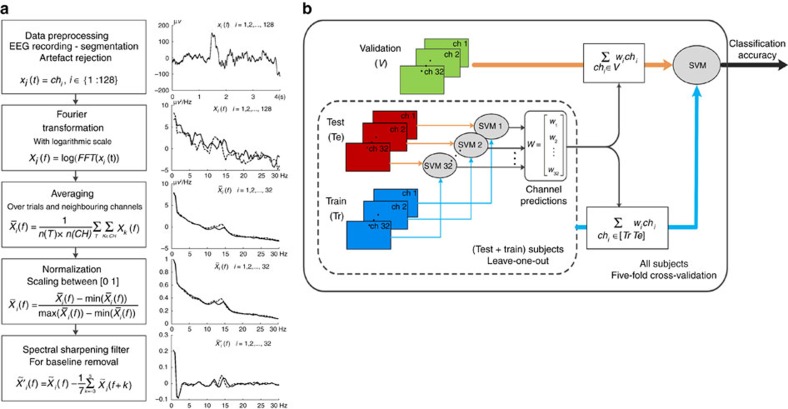
Data preprocessing and MVPC analysis. (**a**) After artefact rejection, data from the remaining 4-s trials of 128-channel sleep EEG data was frequency transformed. To reduce the dimensionality of the data and to increase the signal-to-noise ratio, spectra were averaged over trials and neighbouring channels. Next, spectra of all channels were normalized separately to make them comparable, and a spectral sharpening filter was applied to remove the baseline spectrum and enhance differences between neighbouring frequency bins. (**b**) Training data were strictly separated from validation data in all MVPC analyses. Dimensionality of the data was further reduced in a two-step training procedure. Individual channel performance was determined using separate single-channel classifiers. An average of data from all channels weighted by their standalone performance was then used to train a classifier to distinguish between face and house stimulus conditions. Finally, classification was tested on independent validation data.

**Figure 2:**
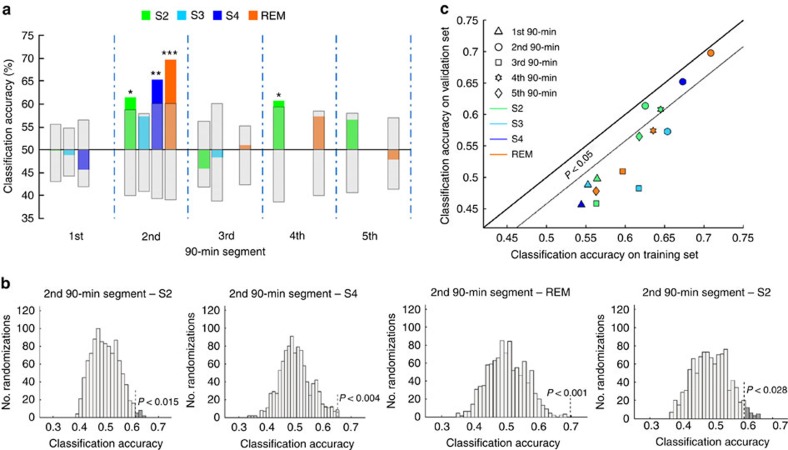
Classification results. (**a**) The content of a previous learning experience can be determined from sleep EEG during the second and fourth 90-min segment of the night. At these times, classification accuracy for all sleep stages is significant or approaches significance. The hatched area shows the 95% confidence interval. Classification accuracies for S4 sleep as well as REM sleep in the second sleep segment remain significant after Bonferroni–Holm correction considering all tests (S4: *P*=0.048, REM: *P*=0.014). (**b**) Significance was assessed using permutation tests to ensure that classification rates are higher than can be expected from data sets with random labelling of the data, that is, not containing any information. To estimate the displayed null-distribution from which exact significance levels of classification results can be determined, the MVPC analysis was repeated 1,001 times on the actual data with randomly shuffled condition labels. Dark grey areas show those randomizations during which classification accuracy on randomly labelled data exceeded accuracy obtained on correctly labelled data. (**c**) If classification accuracies are similar between the training and validation sets, generalizable information could be extracted and the classifier was not overfitted on the training data set. This was the case for all analyses that were significant, that is, for data from the second (circles) and fourth (stars) 90-min segments of the night. Here patterns detected in one set of subjects during classifier training can be generalized to data from a new set of subjects. Data from the first (triangles) and third (squares) 90-min segments show low training accuracy and low accuracy on validation data, indicating that the classifier could not extract information about previous learning content from these periods of the night. *P<0.05, **P<0.01, ***P<0.001.

**Figure 3:**
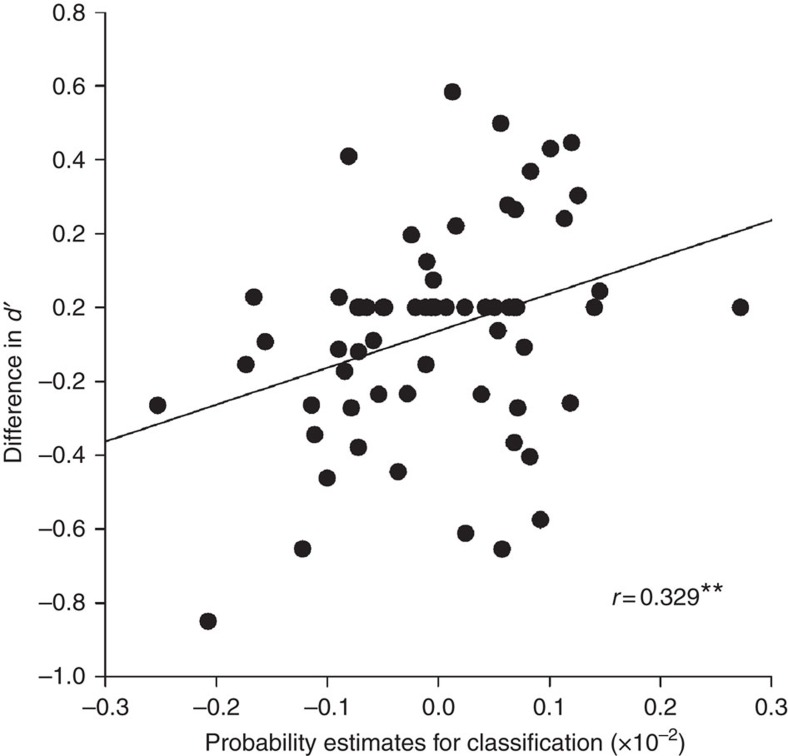
Correlation between classification probability estimates and overnight memory consolidation during SWS sleep. The more confident the classifier was in placing each subject in the correct condition, the more positive the change in memory performance during later recall. Spearman's rho is reported. **P<0.01.

**Figure 4:**
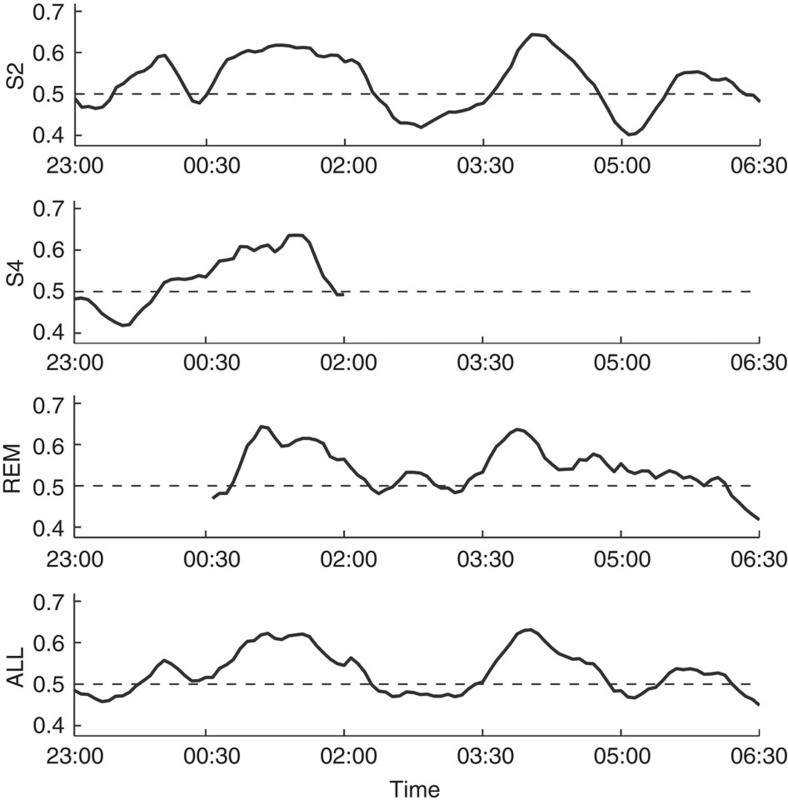
Time course of classification accuracy across the night. Separate analyses were performed for sleep stages S2, S4 and REM sleep. Classification performance follows an oscillatory pattern and peaks around 3 and 6 h after learning in all stages. Timing therefore is more relevant to when memory reprocessing occurs than sleep stage.

**Figure 5:**
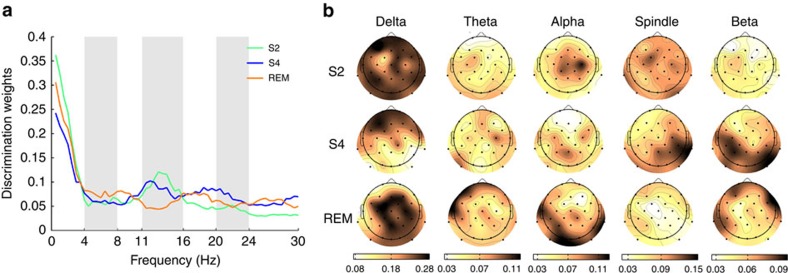
Frequency contributions to memory reprocessing in NREM and REM sleep. (**a**) Discrimination weights show that in NREM sleep stages S2 and S4 spindle activity in the frequency range between 11 and 16 Hz is predictive for learning content. In REM sleep, theta, alpha and higher beta frequencies contributed more to correct classification. Slow frequencies <4 Hz were informative in all sleep stages. (**b**) The topography of predictive channels clearly differs between NREM and REM sleep. In NREM sleep stage S2, mainly delta and spindle frequencies contributed to correct classification. Similarly, frontal delta power and right parieto-temporal spindle activity were most informative for classification during NREM sleep stage S4, together with posterior higher frequency activity. REM sleep shows a more complex pattern. Here slow oscillations of central electrodes and frontal and temporal theta as well as occipital alpha contributed most to discrimination between learning conditions.

Brain activity in REM and NREM sleep is not alike. It is thus reasonable to also assume that information processing in these states will take different forms. To investigate this, the relative contribution of each frequency band to classification can be assessed in terms of classification weights and compared between sleep stages (Fig. 5). Our results show that the frequencies that are important for identifying previous learning content differ between sleep stages. Activity in the range of sleep spindles (11–16 Hz) can distinguish previous learning conditions only in NREM sleep (Fig. 5a). Theta-band activity (4–8 Hz), on the other hand, has higher discriminative power in REM sleep. Slow frequencies <4 Hz were informative in both NREM and REM sleep, but their topographies differ (Fig. 5b). Although there is some resemblance between the feature weight plots and power spectra of sleep, it has to be noted that the feature weights do not follow the typical 1/*f* logarithmic decrease of EEG power spectra but remain essentially constant after a linear decrease in delta frequencies. Moreover, actual classifier input was not the power spectra but the preprocessed data seen in the lower panel of Fig. 1a.

## Discussion

We show that memory processing of a single memory task occurs during all stages of sleep. Reprocessing in REM and NREM sleep, however, has different effects on later memory performance. Although a large number of studies in rodents have observed the occurrence of spontaneous memory reactivation during NREM sleep^4^,^5^,^6^,^19^,^20^, linking this reactivation with improvements in behavioural performance has remained a challenge. Contrary to rodents, task difficulty and training time can be easily adjusted in studies on humans, giving greater power to analyses on behavioural effects. It has early been suggested that memory reactivation during sleep has functional significance for strengthening new memories^21^. Indirect evidence for this assumption has accumulated over the past years^10^,^11^,^22^,^23^,^24^. A recent study in rats found that sleep-dependent reactivation of neurons involved in a simple motor learning task is associated with changes in the task-related spiking behaviour of the same neurons^8^. In this way, reactivation may be related to later improvements in performance. We now show that content-related reprocessing of declarative learning material during NREM sleep influences later memory strength in humans. Conversely, memory reprocessing during REM sleep does not show this graded relation with overnight memory retention.

A number of animal studies detected reactivation of learning activity also in REM sleep^25^,^26^, yet empirical evidence for this has remained ambiguous. We find that memory content is reprocessed during both NREM and REM sleep. The differential significance of memory reprocessing for behavioural performance between these states points towards at least two different mechanisms underlying memory reprocessing during sleep.

Already early on, it has been suggested that memory is formed in a two-stage process. Labile memory traces are formed during exploratory behaviour, when theta power is high. Later, during rest or sleep, long-lasting traces are formed^9^,^21^. Similarly, it has been proposed that, during sleep, slow-wave-related NREM activity and theta-related REM activity have complementary, mutually dependent functions^27^. We find that reprocessing occurs in both NREM and REM sleep. Interestingly, we can demonstrate a correlation between reprocessing and later memory performance only for NREM sleep. This supports the view that reprocessing during REM sleep and NREM sleep serves distinct functions. Our finding is in line with previous studies, which show no behavioural benefit of reactivating memories by cueing during REM sleep^10^. Interestingly, memory replay observed during REM sleep has also been shown to have different characteristics than that in NREM sleep, including a smaller time-compression factor, which is less suited for the induction of long-term potentiation^20^,^25^.

A number of recent studies stress the importance of light NREM sleep, SWS or REM sleep for memory consolidation, respectively^2^,^27^,^28^. Based on these findings, theoretical accounts have suggested that NREM and REM sleep may interact during memory consolidation, emphasizing different aspects of this process. The sequential hypothesis of sleep stresses that different sleep stages have to occur in succession to effectively influence memory function. It assigns specific and substantially different but interdependent roles to NREM and REM sleep regarding the processing of memories^29^. Other accounts suggest that the different processes contributing to memory processing during NREM and REM sleep are separate and independent. Thus the function of NREM and REM sleep in consolidation is assumed to pertain to different aspects or forms of memory^30^. We find that relevant activity occurs in close temporal proximity over different stages and that a single memory task triggers learning-related activity in both NREM and REM sleep EEG. It therefore seems possible that both sleep stages cooperate in the processing of memories. The differential function of NREM and REM sleep stages is still controversial^7^,^16^,^31^. One recent hypothesis is that cortical activity and long-range connectivity differs between sleep stages, allowing local memory reactivation and potentiation in SWS, and network-wide information integration in REM sleep^32^,^33^. This view fits with our findings.

Our data indicate that memory processing in sleep is cyclic in nature and its occurrence might depend more strongly on timing than on the stage of sleep. Instead of occurring in SWS throughout the whole night, reprocessing was detected in S2 and S4 as well as REM sleep in the second 90-min period but not in the first or third. Whether this consolidation window depends on time after learning, time after sleep onset or circadian rhythm cannot be determined in the present study, because these were not varied independently.

Because reprocessing peaks during distinct times of the night, it is unlikely that the detected activity simply reflects ongoing reverberation of learning-related activity or selective fatigue in the involved brain areas. Instead, it must reveal a process that is selectively initiated at specific points during sleep. The finding that reprocessing is the strongest around 3 and 6 h after learning fits well with experiments that found critical periods during memory consolidation, during which memory is particularly sensitive to disruption^34^. Thus inhibiting protein synthesis 15 min and 3 h after learning, but not 1 h after learning, impairs hippocampal one-trial avoidance learning^35^. Similarly, in Drosophila, different behavioural memories and corresponding neuronal traces develop during different time windows over several hours after conditioning^36^, a process that has been linked to systems memory consolidation in humans^37^.

Moreover, our finding of discrete periods for memory reprocessing is reminiscent of previously reported ‘sleep windows', that is, times during which sleep has to occur after learning to strengthen memory^38^,^39^. Along the same lines, Stickgold *et al*.^40^ have found that, for consolidation of a visual discrimination task, mainly duration of SWS and REM sleep in the first and the last quartile of the night, respectively, are most critical parts of the night. Although that task presumably does not rely on hippocampal memory reactivation and might therefore follow a different temporal trajectory, the similarities suggest the possibility of a common mechanism. Further behavioural, electrophysiological and molecular investigations are required to elucidate this underlying mechanism. Moreover, it has still to be ascertained whether the other periods of the night have memory-related functions that cannot be detected by our method.

Because the amount of signal related to memory reprocessing across the whole night is very small compared to the unrelated noise, we used MVPA, which is a very sensitive method to detect systematic differences between large sets of data. However, multivariate approaches are not better suited to supply information about univariate hypotheses than classical tests. Using feature weights and individual channel accuracies (Fig. 5) can, to some extent, illustrate the features that are carrying relevant information. However, these features must be seen within the entire pattern. The following discussion of individual physiologic features should therefore be seen as a starting point for studies focussing on a smaller feature search space.

When looking at the frequencies contributing to correct classification, we find that spindle activity during NREM sleep contributes to the distinction of previous learning conditions. This is consistent with the fact that sleep spindles increases after learning^41^ and correlate with performance^42^. Parietal sleep spindles accompany task-specific reactivation seen in fMRI^43^. Moreover, frontal slow waves, as they appear in our analysis for NREM sleep, have previously been shown to correlate with performance gains observed after memory reactivation induced by cueing during sleep^44^.

Apart from confirming that learning-related information resides in frequency bands that have previously been implicated in memory consolidation, such as NREM spindles and slow oscillations, our results hint at promising objects for future study. We suggest that particular attention should be given to the function of REM sleep theta. Frontal theta power increases during successful memory encoding and retrieval, and theta is also involved in memory processing during wakefulness, such as in controlling, maintaining and storing memory content^45^. Theta has been linked to memory and sleep for a long time, but has only recently received renewed attention^16^,^46^. For instance, theta band activity during sleep has been shown to support formation of imprinting memory in chicks^47^. In humans, another recent study found increased frontal theta power after presentation of cues related to a verbal learning task during sleep^44^,^48^. Moreover, frontal theta in REM sleep is predictive of successful dream recall^49^. These findings stress the active role of theta activity in memory reprocessing during sleep.

It is difficult to demonstrate reactivation directly in humans. Electroencephalographic activity during sleep differs greatly from that during wakefulness in both the time and the frequency domains. Thus amplitude changes over time, as well as power spectral density, cannot be compared between these states. This is owing to different modes of generation and transmission of electrical activity during sleep^50^,^51^. Previous data have shown that reactivation can be both time compressed as well as changing in location (for example, neocortical replay following hippocampal activity)^19^,^52^. Markers reflecting reactivation of neuronal firing patterns observed during learning can thus be altered by a large number of operations, which renders the search space virtually infinite. Because this makes wake-to-sleep classification problematic, and a within-subject design would have to rely on between-session classification that is confounded by various session differences (for example, recording artefacts), we instead opted for a between-subject classification approach. This allowed us to detect information pertaining to a previous learning experience in data recorded in the same state of consciousness. Previous attempts to observe memory reactivation during off-line periods succeeded in showing memory reprocessing during wakefulness but not during sleep^53^,^54^,^55^. Using an approach that trains and tests the classifier in the same state of consciousness made it possible for us to observe material-specific memory reprocessing during sleep and study its dynamics and relation to later behavioural performance.

We used MVPC to decode the content of a previous learning experience from electrical brain activity during sleep. By linking brain activity during sleep with the content of previous learning, our findings bridge studies from multicell recordings in animals, which show learning-related reactivation, to human imaging studies, which show reactivation of brain regions during sleep. Pattern classification methods are powerful tools for investigating the covert mechanisms that link electrical brain activity and behaviour and can thus contribute to our understanding of these complexities.

## Methods

### Subjects

In this study, we recorded EEG data from 32 healthy subjects with no history of neurological or psychiatric disorders. All participants were students, between 18 and 30 years, native German speakers and non-smokers. They were right handed as measured by Edinburgh Handedness Inventory test^56^. Chronotype was assessed via the Munich Chronotype Questionnaire^57^ and experimental timing was adjusted to participants' usual sleep times (sleep midpoint 03:56 h±01:33 h (mean±s.d.)). Subjects were regular sleepers with a habitual sleep duration of 6–9 h. They did not report any chronic or acute sleep-related problems in an initial interview. Moreover, they did no shift work and did not change time zones in the 6 weeks leading up to the experiment. Participants were told to refrain from drinking alcohol, coffee and tea on the days of the experiment and did not take any drugs that affect the central nervous system. All experimental procedures were approved by the local ethics committee (Department of Psychology, Ludwig-Maximilians-Universität München). Informed consent was obtained from all subjects.

### Experimental design

Participants slept in our laboratory on three different nights. The first of these served as an adaption night, to accustom subjects to the environment and to sleeping under the experimental conditions (for example, wearing an EEG cap). In the subsequent two experimental nights, subjects completed an intensive image learning task, during which they studied pictures of either faces or houses. For an exemplary subject, learning took place from 2030 to 2200 hours after the EEG electrodes had been attached, and memory was tested immediately afterwards. The subject then went to bed at 2300 hours for an 8-h sleep period. Memory was tested once more in the morning. The times of the experiment were advanced or delayed such that time to bed corresponded to the individual habitual bedtime of the participants. All subjects participated in two experimental nights, each time learning only one type of images, in a counterbalanced fashion. The two nights were spaced at least 5 days apart. Sleepiness was tested with a visual analogue scale in the evening and after sleep in the morning (Supplementary Table 4).

### Learning task

Subjects studied a set of 100 images of faces or houses in 30 repetitions. Pictures were shown in random order and individual images were always presented in one of the four quadrants of the screen. Participants had to remember the individual pictures and learn to associate the images with the quadrant in which it was presented. Participants were tested once immediately after learning and again in the next morning after a full night of sleep. During both immediate and delayed testing, 100 learned images were presented together with a set of 50 new images in random order. Participants first had to indicate via keypress whether they had seen the image before (with left hand on main keyboard: 1—sure, 2—probably, 3—probably not, and 4—surely not. Responses 1 and 2 were counted as a ‘yes' response, responses 3 and 4 were counted as a ‘no' response). For ‘yes' responses, also the quadrant in which the image had been presented was probed (with right hand on numerical pad: 1—lower left, 3—lower right, 7—upper left, and 9—upper right). Image material was derived from two different sources: 300 pictures of houses were taken from German online real estate sites, 300 pictures of neutral faces were taken from Minear and Park^58^.

This task was chosen because it is a declarative task that is supposed to involve the hippocampus, and sleep-related reactivation has mainly been shown in the hippocampus^10^,^19^. Face and house processing are clearly different in event-related EEG potentials and fMRI^59^. Face processing activates the mid-fusiform gyrus (fusiform face area) and the occipital face area in the occipito-temporal cortex as well as other temporal areas^60^, whereas processing of houses activates the parahippocampal place area and the lateral occipital gyrus^61^,^62^.

### EEG recording

Sleep EEG was recorded using an active 128 channel Ag/AgCl-electrode system (ActiCap, Brain products, Gilching, Germany) with 1 kHz sampling frequency and a high-pass filter of 0.1 Hz. Electrodes were positioned according to the extended international 10–20 electrode system. For sleep scoring, recordings were split into 30-s epochs and sleep stages were determined on electrodes C3/C4 according to standard rules by two independent raters^63^. Average sleep durations are reported in Supplementary Table 5.

### Methodological considerations

One of the challenges in sleep research is the difficulty of recording large sample sizes and the large amount of data that is recorded. The goal of classical analyses, which use multiple univariate comparisons (for example, classical fMRI analysis), is to find single features that are strong enough independently to distinguish between conditions. Such features are unlikely to exist in high-density all-night EEG recordings, which thus present a problem better addressed by a multivariate approach. In multivariate analyses, it is of interest whether the overall pattern of data contains information that is relevant to distinguish conditions. A prominent method that can deal with large numbers of data dimensions is MVPC. However, high-dimensional, low-sample-size data, such as EEG recordings, pose specific problems for classical statistical testing as well as for MVPC^64^,^65^. For this kind of data, it is important to minimize the number of features. If the signal across features is highly correlated, as in EEG data, this can be achieved by averaging, which reduces dimensionality of the data and at the same time increases signal-to-noise ratio. We developed a two-step procedure that uses spatial averaging and a channel-based weighted average to improve classifiability of our data (Fig. 1). These steps are described in detail in the sections ‘Data preparation' and ‘Multivariate pattern classification' below.

### Data preparation

For artefact rejection and further analysis, EEG data were split into 4-s trials. Artefact rejection was done in a semiautomatic process using custom MATLAB scripts. Based on the distributions of different parameters of the raw data and power spectrum, rejection thresholds were chosen for each recording individually to make sure that only a minimal number of artefacts remained in the data. We tested for disconnected electrodes (outliers in overall spectral power), sudden jumps of the signal (outliers in amplitude changes) and muscle artefacts (outliers in spectral power between 110 and 140 Hz). Outlier thresholds were automatically suggested based on the variance of the data and manually confirmed upon visual inspection of parameter distributions and of the raw data. Trials containing artefacts were removed from the data set; channels that contained too many trials with artefacts were removed entirely and interpolated using routines provided by EEGLAB^66^. Whether individual epochs or channels were to be removed was determined automatically so that data loss was kept minimal. Artefact-free trials were then transformed into the frequency domain using Fourier transformation. To obtain smooth spectra, Welch's method was used for this, averaging over 10 Hamming windows of 2-s length with 95% overlap, resulting in a final data resolution of 0.5 Hz. Data were used up to a maximum frequency of 30 Hz.

The subsequent steps for data preparation were implemented to (1) increase signal-to-noise ratio, (2) reduce dimensionality of the data and (3) adapt the signal for between-subject classification. First, we averaged power spectra across electrodes within a radius of approximately 3 cm around the 32 evenly spread locations of the extended 10–20-system to decrease the number of redundant features and increase signal-to-noise ratio as well as spatial similarity between subjects. We then separately averaged over all artefact-free trials available for each 90-min segment and sleep stage, to obtain a reliable estimate of spectral properties. This also ensures that an equal number of epochs per subject enters analysis, which is important for classification to remain unbiased. To remove amplitude differences between channels, which are caused by the distance of each channel to the reference electrode, spectra of all channels were separately normalized between zero and one. This also removed between-subject variability in general spectral power.

Because baseline EEG power spectra are highly similar and differences between conditions can be expected to be of smaller magnitude, these differences need to be enhanced within the spectra. We thus applied a spectral sharpening filter, which removes the baseline spectrum and emphasizes differences between neighbouring frequencies in a final preparation step. To achieve this, we subtracted a moving average of six neighbouring frequency bins (window size: 3 Hz) from the signal. This accentuates the smaller differences in power between frequencies within the spectrum. This is a valid procedure because neighbouring data points in the power spectrum represent neighbouring frequencies from the same signal and are therefore strongly correlated.

Subjects were only included in the analysis if they had at least 40 artefact-free trials within the respective sleep stage and segment (that is, 160 s of data). Only segments and stages with at least 11 subjects were analysed. The number of subjects and trials available for each 90-min segment and sleep stage can be found in Supplementary Table 6. As can be seen from that table, the amount of data available was unrelated to classifier performance.

### Multivariate pattern classification

In the present study, we tested whether electrical brain activity during sleep holds information about the content of previously learned visual stimuli. Instead of the typically used multiple univariate tests, we employed a multivariate classification approach, which can detect information contained in the overall pattern of brain activity, but is not distinguishable from single features.

Sleep EEG recordings from 64 nights (32 subjects, two conditions each) were analysed using a classification algorithm developed on the basis of linear support vector machines (SVM). The aim was to detect material-specific information in the data. Please note that, whereas the experiment followed a within-subject design, classification was done between subjects, with both nights of each participant (face and house conditions) assigned either to the training, test or validation set. All analyses were done with the Matlab implementation of libsvm 3.1 (http://www.csie.ntu.edu.tw/~cjlin/libsvm). EEG recordings pose problems typical of high-dimensional, low-sample-size data (potential feature space of 128 channels times 60 frequency bins). We thus preprocessed the data to reduce the number of features and increase signal-to-noise ratio (see Fig. 1 and ‘Data preparation' section), averaging over neighbouring channels to lower the number of channels to 32. To further enhance relevant features, we used a stepwise procedure for classification, which first regarded every channel as an independent classifier and then combined outcomes of this first step for the final analysis.

We split data into independent training and validation sets. In a first step, one linear SVM was trained for each of the 32 averaged EEG channels on all but one subject of the training set to see how much each channel contributes to distinguishing the content of learning conditions (‘face' learning or ‘house' learning). This channel-based classification was cross-validated in a leave-one-out procedure on each subject, and the obtained classification accuracies were averaged over all cross-validation runs. In the second step, this average classification accuracy from each channel was used as a weight to obtain a weighted average of the 32 channels. The main SVM was then trained on this weighted training set and classification accuracy was tested on the independent validation set. The main reason for weighted averaging of channels was to reduce feature space dimensionality, because feature weights cannot be reliably determined if sample size is much smaller than the number of features^67^. Apart from this, weighted averaging can amplify relevant information in the data. This two-step classification process was cross-validated on independent data using 280 repetitions of a 5-fold procedure, which covers the whole data set with five independent validation sets.

We used permutation tests to assess significance. These tests sample the distribution of the null hypothesis by random shuffling of the original data, which is repeated a large number of times. To obtain the correct null-distribution for our data, we randomly shuffled condition labels, that is, the two conditions of each subject were randomly labelled as ‘face'/‘house' or as ‘house'/'face', effectively removing all relevant data pertaining to the effect of interest, while keeping other dependencies in the data constant. We then calculated classification accuracies for the randomly labelled data to estimate the random distribution. This was repeated 1,001 times. Significance was calculated by determining the percentage of times that classification on randomly labelled data produced accuracies that were equal to or higher than the classification accuracy obtained from the actual data. If randomly labelled data did not result in a classification accuracy equal to or higher than the actual data, then the *P* value was determined by the number of random repetitions that were calculated (see Supplementary Fig. 1).

To assess whether reprocessing occurs uniformly across time, we split the night, starting from time to bed, into five 90-min segments, which are likely to include a whole sequence of sleep stages (S2, S3, S4 and REM sleep; see Supplementary Table 5 for details of sleep stage distribution). In this first analysis, we classified separately for all segments and sleep stages to assess the temporal dynamics of memory reprocessing. To determine a more fine-grained time course of classification accuracy, we moved a sliding window with a width of 22.5 min in steps of 4.5 min across the night. We then estimated classification accuracy within each window using the same two-step classification procedure as before. Analysis was done separately for each sleep stage and the same inclusion criteria were applied as in the main analysis.

To assess which features of the sleep EEG are particularly predictive, we analysed classification weights. To assess which features of the sleep EEG are particularly predictive, we analysed classification weights. The absolute value of the weights are informative about how much each frequency band and channel contributes to successful distinction. We averaged the classification weights over all repetitions of the training procedure, resulting in an averaged 32 (channels) × 60 (frequency bins) weight matrix. To examine frequency contributions to memory reprocessing, we further averaged the absolute values of these weights over all channels (see Fig. 5a). The topography of predictive channels (see Fig. 5b) was obtained by averaging absolute values of classification weights for each channel over different frequency bands (delta: 0.5–3.5 Hz, theta: 4–7.5 Hz, alpha: 8–10.5 Hz, spindle: 11–15.5 Hz, beta: 16–30 Hz). We chose to analyse classification weights for frequencies obtained in the inner train–test loop (Fig. 1) because they can give additional information on the topography of predictive channels. These frequency weights are confirmed by weights from the outer validation loop (Fig. 1). Frequency contributions to classification assessed from both loops show the same pattern (see Supplementary Fig. 2).

### Behavioural performance

For assessment of memory performance, we calculated the memory sensitivity index *d*′ as the difference of *z*-values between correctly recognized old items versus falsely recognized new items (*z*(hits)−*z*(false alarms)). Performance presleep and postsleep, as well as memory consolidation across the nights is reported in Supplementary Table 1. We correlated overnight memory consolidation with time spent in different sleep stages (see Supplementary Table 2). To examine whether memory reprocessing during sleep is associated with better memory performance, we correlated the probability estimates for classification given by the classifier with overnight memory consolidation measured as the difference between postsleep and presleep *d*′ values. No such correlation was found for encoding or retrieval performance per se (see Supplementary Table 3). For each subject, results of all 280 repetitions of the 5-fold cross-validation procedure were averaged. We conducted this analysis separately for different sleep stages. All correlations report Spearman's rho.

### Data availability

All data and codes are available from the corresponding authors upon request.

## Additional information

**How to cite this article:** Schönauer, M. *et al*. Decoding material-specific memory reprocessing during sleep in humans. *Nat. Commun.* **8**, 15404 doi: 10.1038/ncomms15404 (2017).

**Publisher's note:** Springer Nature remains neutral with regard to jurisdictional claims in published maps and institutional affiliations.

## Supplementary Material

Supplementary Information Supplementary Figures and Supplementary Tables
