# Supplementary material for: Decoding material-specific memory reprocessing during sleep in humans
Source: Nat Commun. 2017 May 17;8:15404. doi: 10.1038/ncomms15404 (PMC5442370; doi:10.1038/ncomms15404)
Supplement: Supplementary Information — Supplementary Figures and Supplementary Tables [file ncomms15404-s1.pdf]

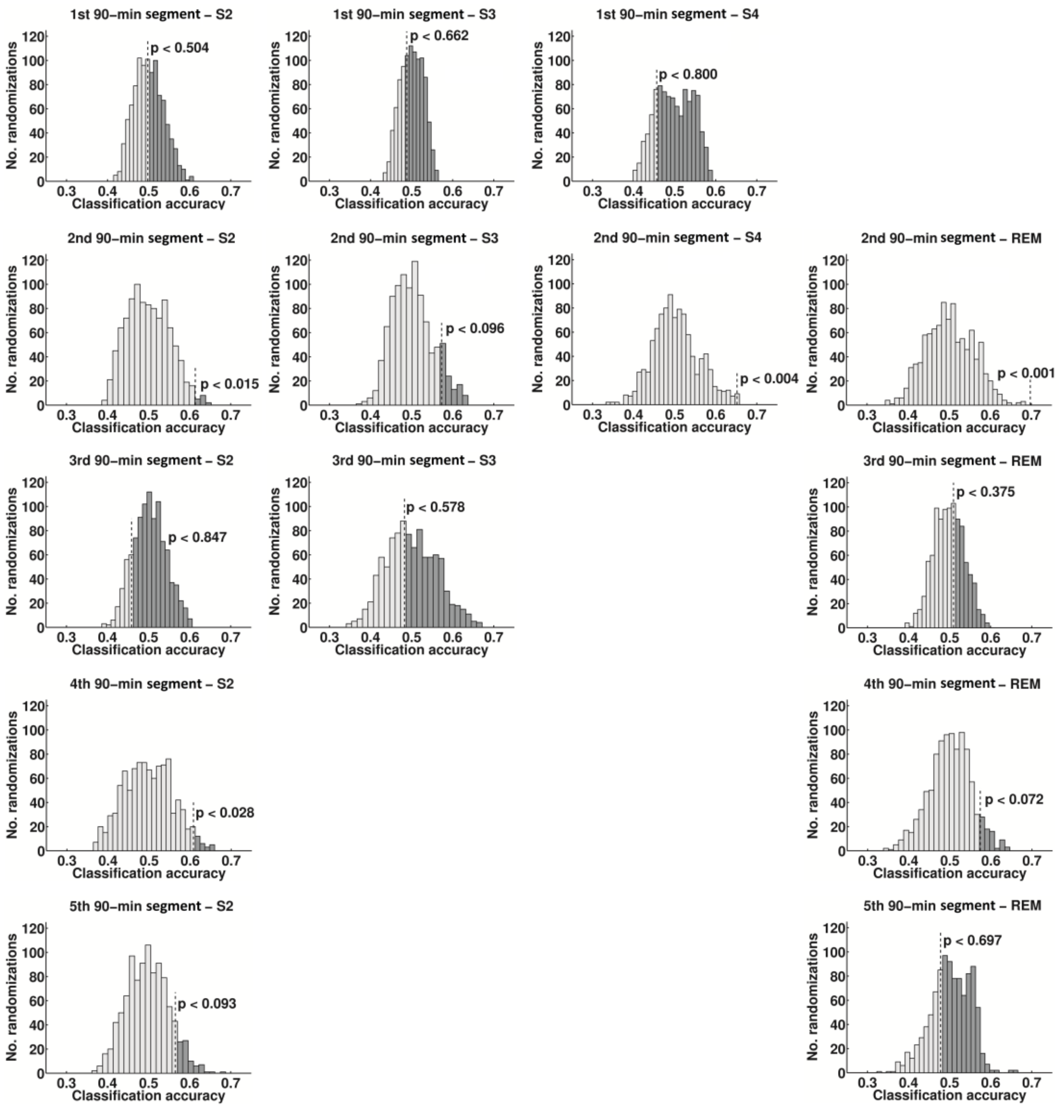


**Supplementary Figure 1.** Randomization statistics for classification in all segments (rows) and sleep stages (columns). Dark grey areas indicate those randomizations where classification accuracy for randomly labeled data exceeded the classification accuracy obtained with correctly labeled data.

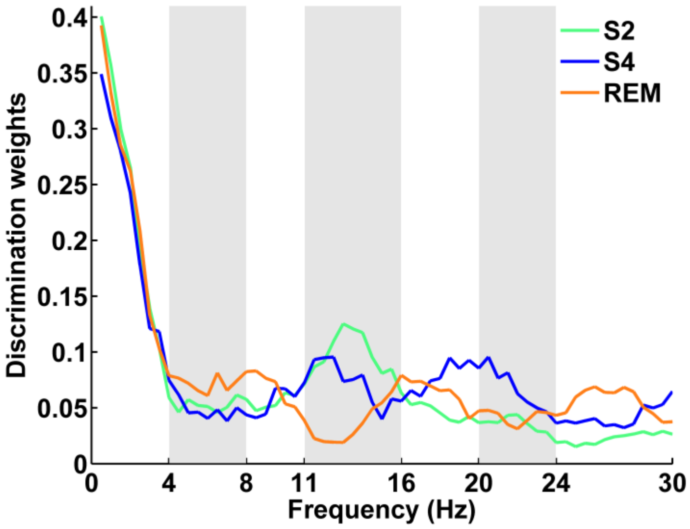


**Supplementary Figure 2.** Absolute classification weights for the outer loop SVM. Note that weights estimated in the outer loop closely resemble those obtained in the inner loop of the two-step classification procedure (Figure 5).

**Supplementary Table 1.** Memory sensitivity d' in the face and house learning conditions

| | pre | post | difference | p-value |
|---|---|---|---|---|
| **Face pictures** | 3.72 ± 0.12 | 3.66 ± 0.12 | -0.07 ± 0.04 | 0.116 |
| **House pictures** | 3.42 ± 0.13 | 3.34 ± 0.14 | -0.08 ± 0.05 | 0.167 |

*Values are given as mean ± SEM. Two sided t-test for dependent measures is reported. Note that no significant forgetting occurred across the night.*

**Supplementary Table 2.** Correlations between total time spent in sleep stages and memory consolidation (difference in d' post-pre) over sleep for all available nights

| | ***r*** | ***p*** | ***n*** |
|---|---|---|---|
| **S2** | -0.139 | 0.272 | 64 |
| **S3** | 0.106 | 0.405 | 64 |
| **S4** | **0.254*** | **0.043** | 64 |
| **REM** | -0.048 | 0.707 | 64 |

**Significant two-sided test at threshold of α < 0.05; Spearman's rho is reported.*

**Supplementary Table 3.** Correlations between classifier performance (probability estimates for classification) and memory consolidation (difference in d' post-pre) over sleep for all available nights

| | **difference** | | **pre** | | **post** | | |
|---|---|---|---|---|---|---|---|
| | *r* | *p* | *r* | *p* | *r* | *p* | *n* |
| **S2 sleep** | 0.099 | 0.436 | 0.023 | 0.859 | 0.044 | 0.733 | 64 |
| **SWS sleep** | **0.329**** | **0.008** | -0.055 | 0.667 | 0.065 | 0.608 | 64 |
| **REM sleep** | -0.199 | 0.142 | 0.069 | 0.611 | -0.036 | 0.791 | 56 |

*** Significant two-sided test at threshold of α < 0.01; Spearman's rho is reported.*

**Supplementary Table 4.** Levels of fatigue in the face and house learning conditions

| | Face night | House night | p-value |
|---|---|---|---|
| **evening** | 5.3 ± 2.0 | 5.5 ± 1.8 | 0.772 |
| **morning** | 3.7 ± 1.9 | 3.6 ± 1.6 | 0.924 |

*Values are given as mean ± SD. Participants were asked to rate their sleepiness on a visual analogue scale with the end points 0 (not tired at all) and 10 (very tired). Two sided t-test for dependent measures is reported.*

**Supplementary Table 5.** Sleep data (mean ± SD)

| | W | S1 | S2 | S3 | S4 | REM |
|---|---|---|---|---|---|---|
| **1st 90-min segment** | 20.3 ± 11.8 | 4.8 ± 2.7 | 29.9 ± 11.8 | 14.2 ± 6.7 | 17.9 ± 13.8 | 2.4 ± 3.3 |
| **2nd 90-min segment** | 3.5 ± 7.8 | 2.1 ± 1.9 | 50.9 ± 12.8 | 11.1 ± 6.6 | 10.0 ± 8.7 | 11.0 ± 6.3 |
| **3rd 90-min segment** | 4.2 ± 10.9 | 2.2 ± 2.0 | 48.5 ± 10.9 | 8.0 ± 5.1 | 5.1 ± 5.7 | 20.3 ± 7.4 |
| **4th 90-min segment** | 6.9 ± 12.8 | 2.7 ± 2.2 | 49.0 ± 13.4 | 5.6 ± 5.1 | 1.8 ± 3.8 | 21.0 ± 8.2 |
| **5th 90-min segment** | 6.9 ± 11.4 | 4.9 ± 3.8 | 42.4 ± 12.0 | 3.3 ± 4.4 | 1.5 ± 4.1 | 26.4 ± 11.2 |
| ***total*** | 48.2 ± 41.5 | 18.7 ± 8.9 | 237.7 ± 40.4 | 42.5 ± 15.0 | 36.4 ± 23.7 | 96.0 ± 23.8 |

*Average sleep latency was 20.1 ± 17.0 min (mean ± SD). Please note that total time does not correspond to the sum of 90-min segment values because participants slept slightly longer than five 90-min sleep segments.*

**Supplementary Table 6.** Number of participants and trials that entered classification in different segments and sleep stages. Only data points with N ≥ 11 and number of trials ≥ 40 for both the face and house learning conditions were entered into analysis in each segment and stage.

| | **S2** | | **S3** | | **S4** | | **REM** | |
|---|---|---|---|---|---|---|---|---|
| | *N* | *trials* | *N* | *trials* | *N* | *trials* | *N* | *Trials* |
| **1st 90-min segment** | 31 | 472 ± 47 | 30 | 355 ± 100 | 18 | 455 ± 84 | 3 | 279 ± 118 |
| **2nd 90-min segment** | 32 | 494 ± 33 | 20 | 321 ± 102 | 12 | 375 ± 93 | 18 | 360 ± 74 |
| **3rd 90-min segment** | 29 | 483 ± 46 | 16 | 300 ± 121 | 6 | 344 ± 111 | 24 | 417 ± 89 |
| **4th 90-min segment** | 24 | 478 ± 53 | 9 | 252 ± 110 | 2 | 257 ± 148 | 19 | 443 ± 59 |
| **5th 90-min segment** | 20 | 454 ± 94 | 0 | | 0 | | 18 | 415 ± 115 |

*Values for total number of trials collapsed over the face and house conditions that entered classification, given as mean ± SD.*
